# Supplementary material for: PerturbNet predicts single-cell responses to unseen chemical and genetic perturbations
Source: Mol Syst Biol. 2025 Jul 10;21(8):960–82. doi: 10.1038/s44320-025-00131-3 (PMC12322087; doi:10.1038/s44320-025-00131-3)
Supplement: Supplementary file 1 — Appendix [file 44320_2025_131_MOESM1_ESM.pdf]

Appendix for PerturbNet predicts single-cell responses to unseen chemical and genetic perturbations

Hengshi Yu[1*], Weizhou Qian[2*], Yuxuan Song[2], and Joshua D. Welch[2,3+]

[1]Department of Biostatistics, University of Michigan, Ann Arbor, MI 48109, USA

[2]Department of Computational Medicine and Bioinformatics, University of Michigan, Ann Arbor, MI 48109, USA

[3]Department of Computer Science and Engineering, University of Michigan, Ann Arbor, MI 48109, USA

[*]Equal contribution

[+]Corresponding author: welchjd@umich.edu

Table of Contents

A. Appendix Supplementary Notes

A.1 Covariate adjustment gives better predictions for PerturbNet **4**

A.2 Notes of stereoisomers in LINCS-Drug **4**

A.3 Notes of comparison to chemCPA **4**

A.4 Notes of comparison to Biolord **5**

A.5 Notes of comparison to GEARS **5**

A.6 Attributing Perturbation Effects to Specific Perturbation Features **6**

A.7 Experimental hardware configuration **7**

B. Appendix Supplementary Figures

Appendix Figure S1 **8**

Appendix Figure S2 **9**

Appendix Figure S3 **10**

Appendix Figure S4 **11**

Appendix Figure S5 **12**

Appendix Figure S6 **13**

C. Appendix Supplementary Tables

Appendix Table S1 **14**

Appendix Table S2 **15**

Appendix Table S3 **16**

Appendix Table S4 **17**

Appendix Table S5 **18**

Appendix Table S6 **19**

Appendix Table S7 **20**

Appendix Table S8 **20**

Appendix Table S9 **21**

Appendix Table S10 **21**

A Appendix Supplementary information

A.1 Covariate adjustment gives better predictions for PerturbNet

Apart from perturbations, several other factors can influence single-cell gene expression states, such as cell type and dosage. Hence, incorporating these important confounders into the model is expected to enhance its predictive accuracy. In the adjusted PerturbNet model, we concatenate the covariates matrix with the perturbation representations $\boldsymbol{Y}$, forming a joint condition representation for the cINN. To verify whether we properly adjust for the covariates, we compared the adjusted PerturbNet with the unadjusted version using the sci-Plex dataset, including cell type (categorical), dosage (continuous), and treatment time (continuous). (**Fig. S3**) demonstrates that the adjusted PerturbNet significantly outperforms the unadjusted version, achieving a higher R^2 for both highly variable genes and DEGs.

A.2 Notes of stereoisomers in LINCS-Drug

We created 5 distinct train/test splits to investigate whether including stereoisomers affects the models' prediction accuracy on unseen drugs. For each test split, we randomly selected 100 drugs with stereoisomers from the LINCS-Drug dataset. We then created two different training sets of 2000 drugs for each split. One training set consisted only of randomly sampled drugs without any stereoisomers. The other training set included all the stereoisomers from the test splits along with randomly sampled drugs without stereoisomers. We trained PerturbNet and chemCPA on these two different training sets and evaluated them using the same testing set for each train/test split.

A.3 Notes of comparison to chemCPA

Built on the CPA framework, chemCPA is an encoder-decoder-based deep learning method that predicts the effects of unseen drugs. The encoder generates a compositional latent space of gene expression, chemical representations, and possible covariates, which the decoder uses to make predictions. For benchmarking, we selected chemCPA with RDKit embeddings because this version achieved the highest R^2 reported in the chemCPA paper.

We thought LINCS-Drug is a good dataset to compare PerturbNet and chemCPA because chemCPA models normalized data while sci-Plex dataset has raw count data. Additionally, in

the chemCPA paper, the authors pretrained chemCPA on the LINCS-Drug dataset and fine-tuned the model on the sci-Plex dataset. The pretrained chemCPA performed better than the version without pretraining. However, this requires the selected genes in sci-Plex to include all genes from the LINCS-Drug dataset. Since we are using different benchmark gene sets, we chose to compare chemCPA only on the LINCS-Drug dataset. We used the default training parameters, automatic early stopping and followed the instructions from `https://github.com/theislab/chemCPA`. The version of chemCPA is 1.0.0.

A.4 Notes of comparison to Biolord

Biolord is a deep generative method for learning disentangled representations in single-cell data and can predict cellular responses to both chemical and genetic perturbations without introducing mutations. We trained Biolord on the sci-Plex Norman dataset for chemical and genetic perturbations separately. Following the instructions from `https://github.com/nitzanlab/biolord`, we used the default parameters and automatic early stopping. The only difference was that we changed the batch size from 32 to 30 on the Norman dataset to resolve training errors.

For benchmarking on the sci-Plex dataset, we used the same covariates as Biolord did in the paper (Piran et al. 2024), which are cell type and dosage. In our benchmarking results, the adjusted PerturbNet outperformed Biolord on the sci-Plex dataset, which appears to contradict the conclusion from the Biolord paper. There are two main reasons for this discrepancy. First, the previously published code for calculating R^2 of PerturbNet was outdated and problematic, because we introduced two additional row and column scaling steps, which significantly reduced R^2. We have fixed this issue in our updated version. Second, throughout the paper we evaluated population-level predictions and did not stratify the predictions by covariates. Biolord evaluations were focused on predictions stratified by covariates, since the paper aimed to disentangle the effects of covariates. The version of Biolord is 0.0.2.

A.5 Notes of comparison to GEARS

GEARS is a deep learning method that uses a graph neural network (GNN) to generate embeddings based on gene co-expression and gene-ontology for perturbations. These embeddings are summed and passed through an MLP, followed by a gene-specific layer that predicts the mean gene expression

induced by multi-gene perturbations. We trained GEARS using the default parameters, following the instructions from `https://github.com/snap-stanford/GEARS`. The version of GEARS 0.0.2.

A.6 Attributing Perturbation Effects to Specific Perturbation Features

Having established that PerturbNet can successfully predict the effects of unseen perturbations, we reasoned that the model could give insights into which specific perturbation features are most predictive of cell state distribution shifts. For example, it would be desirable to know which atoms in a drug or which gene functions most strongly influence the model predictions. Such insights can give hints about mechanisms and help build confidence that the model is learning meaningful relationships between perturbations and cell states. We employ the method of integrated gradients (Sundararajan et al. 2017) to determine, for each feature of a perturbation, whether the presence of the feature increases or decreases the probability of cells being in a particular state. To do this, we divide the space of observed cell states into discrete cell types through unsupervised clustering. Then we train neural networks to classify cell states (including predicted cell states output by the mapping network) into these discrete types (**Fig. EV4A**). For ease of interpretation, we train a binary classifier for each cell cluster, to classify the cells as either belonging to that cluster (1) or not (0). We then use the method of integrated gradients to calculate an attribution score for each feature of an input perturbation. This score tells whether each feature increases or decreases the probability of generating cells of a particular type.

As an example, we performed integrated gradient attribution on the down-sampled LINCS-Drug datasets with representative high quality perturbation data. We performed k-means clustering ($k = 30$) on the latent values of VAEs trained on the LINCS-Drug. (**Fig. EV4A-B**). We then trained neural networks to classify cell latent values into these 30 clusters. For each cluster, we could then calculate an attribution score for each input feature of a perturbation. A positive attribution score indicates that a feature increases the probability of generating cells in that particular cluster, whereas a negative score indicates a decreased probability of generating cells in that cluster.

We visualized the attribution scores for small molecule perturbations by coloring each atom in the molecular structure. Two representative drugs and selected clusters are shown in **Fig. EV4C-D**. We ensured that the selected clusters initially contained cells treated with the representative drugs to prevent the extreme sample bias, such as empty clusters leading to negative attribution

scores. In our examples, nearly the entire structure of “BRD-K27305650” shows a positive attribution score for cluster 11, while most of its substructures contribute negatively to cluster 29. This attribution score pattern suggests that the drug increases the likelihood of driving cells into cluster 11’s states and prevents the states represented by cluster 29. The high cell density in cluster 11 and low cell density in cluster 29 confirm that the attribution scores align with the real data patterns (**Fig. EV4C, bottom**). Another example is “BRD-A19500257”, where the positive attribution scores are more widely distributed across its structure for cluster 29 compared to cluster 20. This pattern is further supported by the observed density difference between the two clusters (**Fig. EV4D, bottom**).

A.7 Experimental hardware configuration

We successfully trained PerturbNet on three different configurations:

(1) NVIDIA Tesla V100 + 2 x 2.4 GHz Intel Xeon Gold 6148

(2) NVIDIA A40 + 2 x 2.9 GHz Intel Xeon Gold 6226R

(3) NVIDIA GeForce RTX 4060 + Intel Core i7-14700HX

All computing jobs were performed within 64GB of RAM limit.

B Supplementary Figures

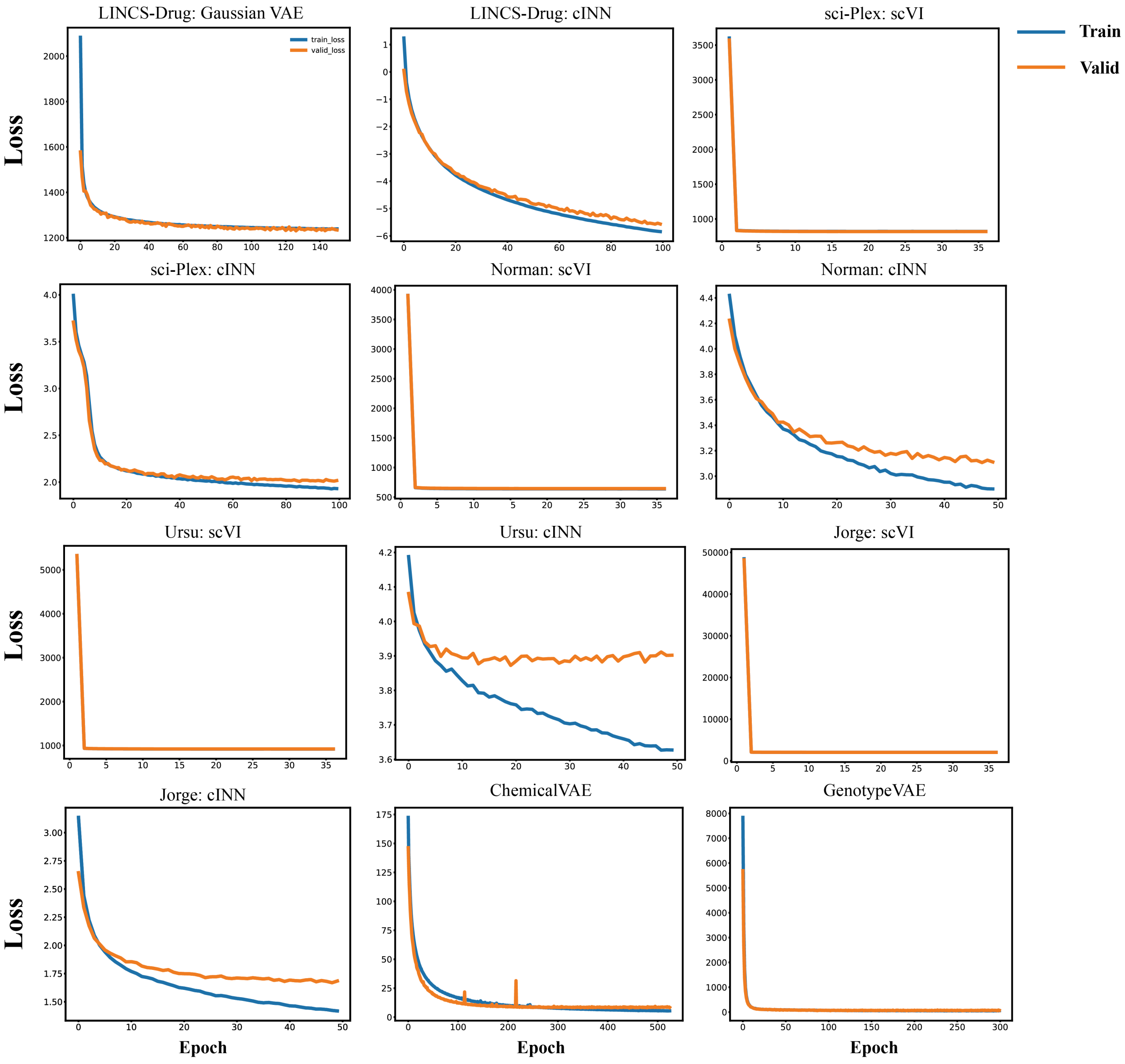

Appendix Figure S1 | Examples of loss curves for PerturbNet modules across various datasets

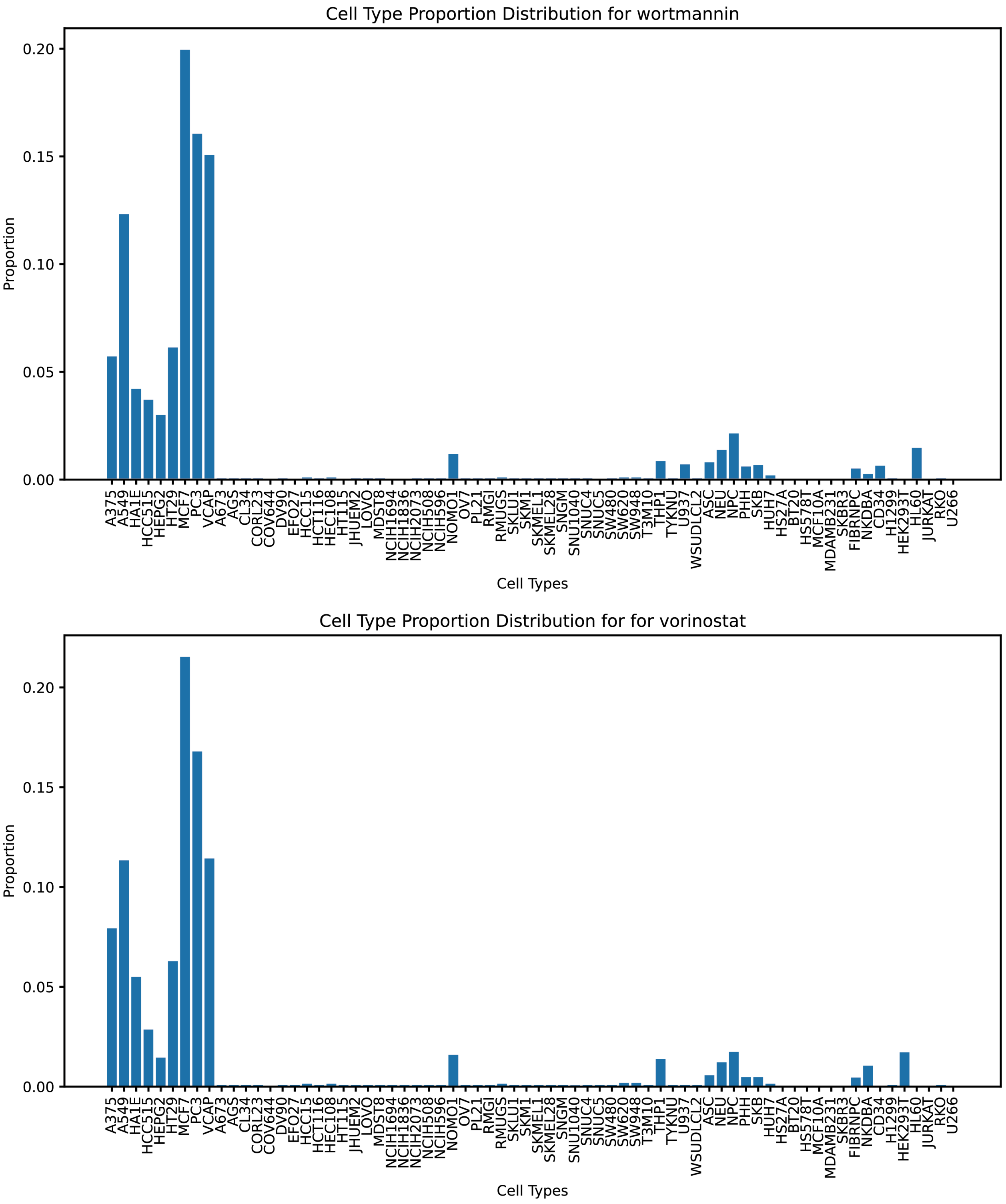

Appendix Figure S2 | Bar plot of cell type proportions for cells treated with the selected drugs in the LINCS-Drug dataset. The cosine similarity is 0.985.

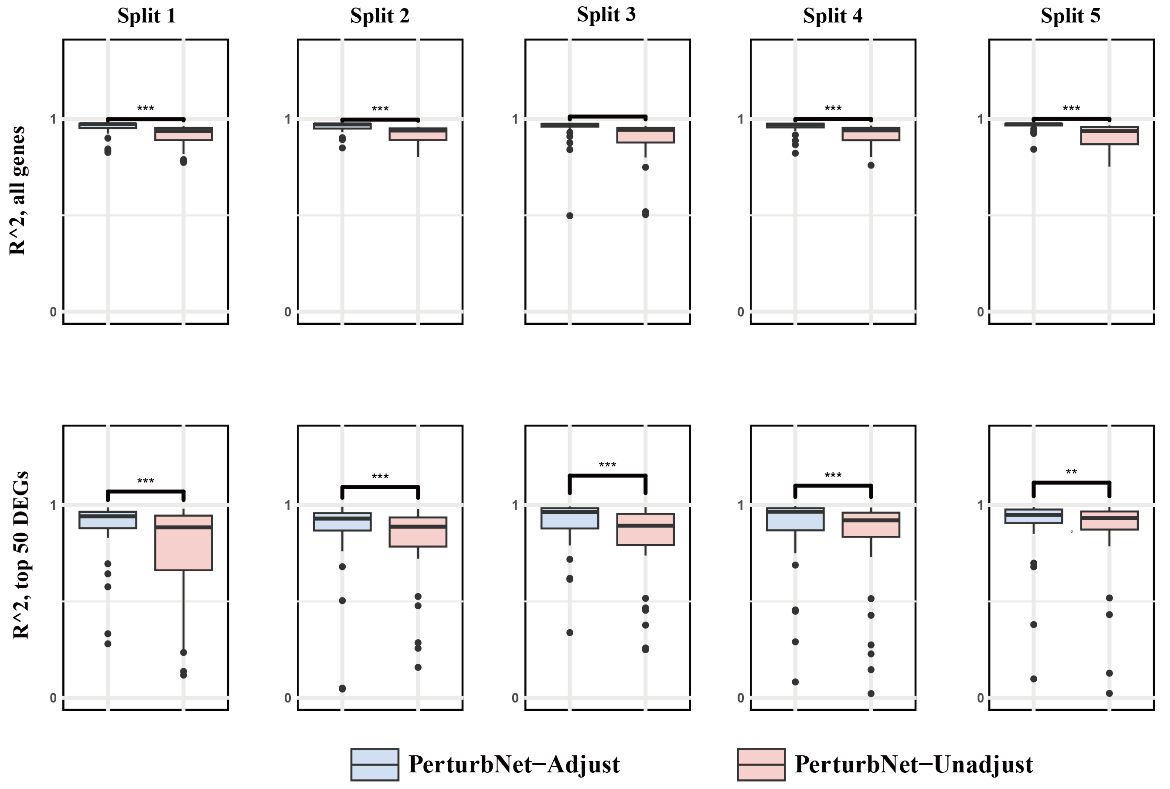

Appendix Figure S3 | Covariate adjustment gives better predictions for PerturbNet Box plots of R^2 calculated on all genes and top 50 DEGs in the 5 test splits of sci-Plex dataset, PerturbNet-Adjust refers to the PerturbNet model with covariates included, while PerturbNet-Unadjust is the version without covariates. Paired one-sided Wilcoxon tests were conducted to compare the performance of the models. The significance of the P-values is denoted as follows: *P < 0.05, **P < 0.01, ***P < 0.001.

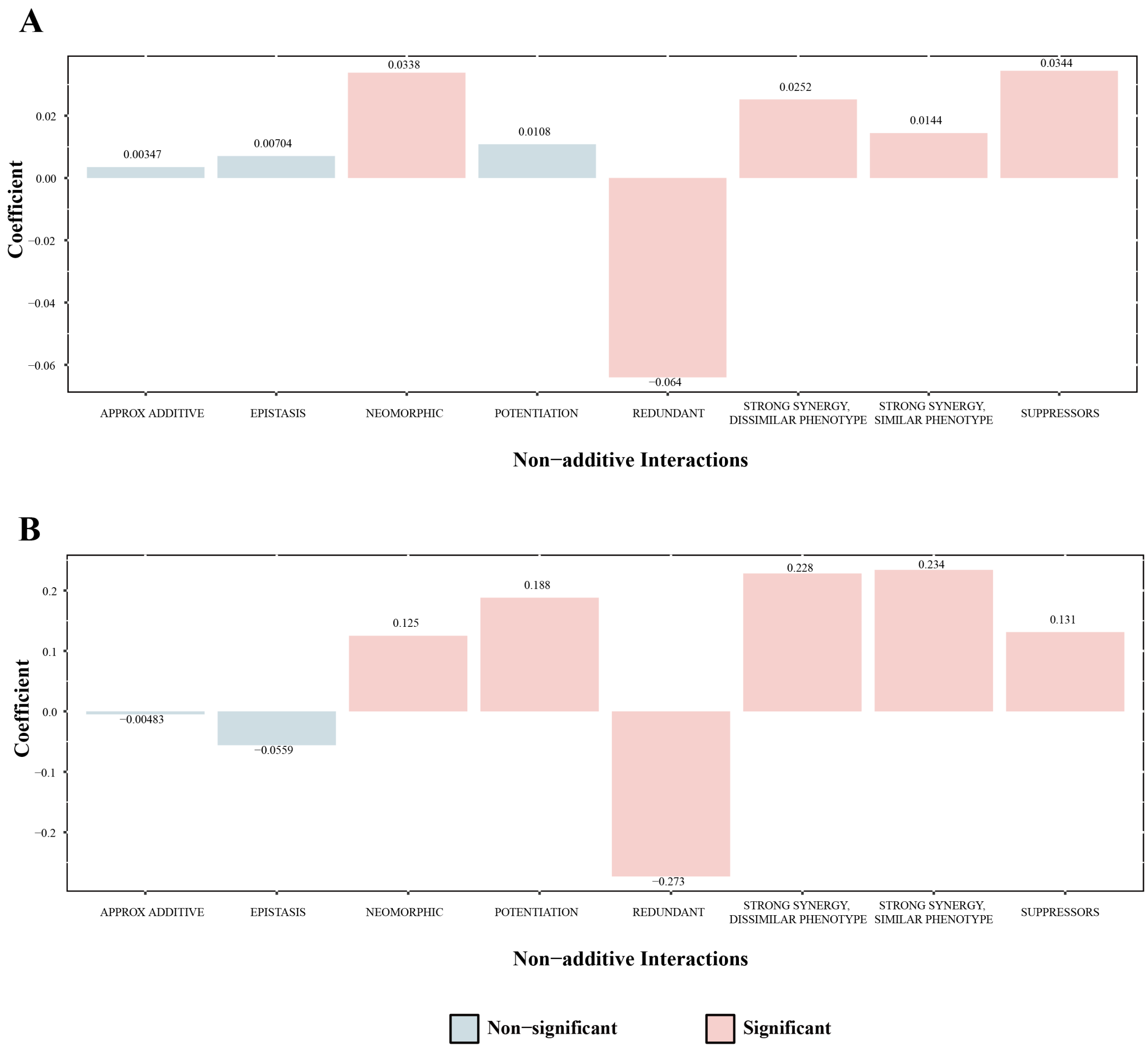

Appendix Figure S4 | Linear regressions test summary for non-additve interactions (A) Linear regression fitted on the R^2 of highly variable genes. **(B)** Linear regression fitted on the R^2 of top 50 DEGs

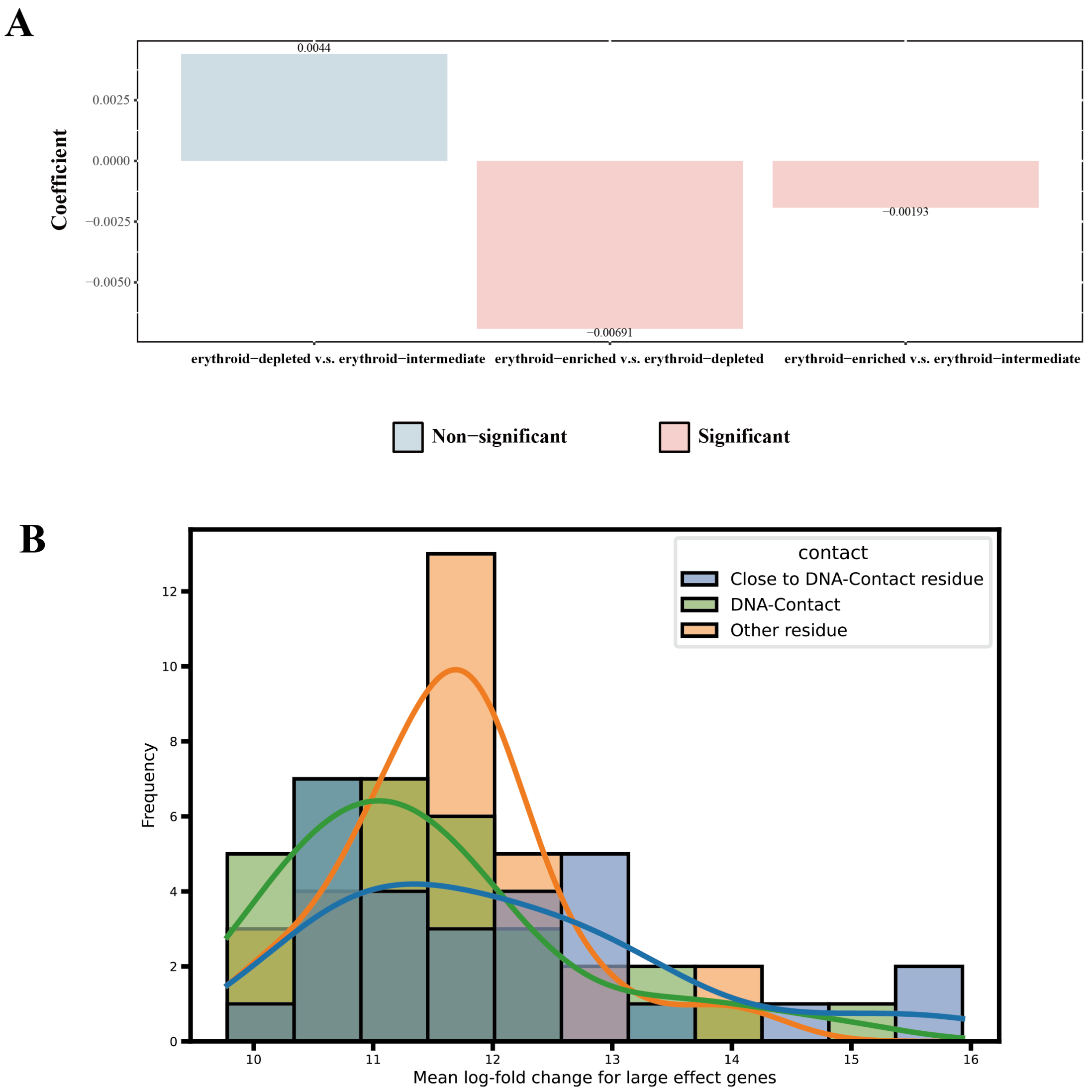

Appendix Figure S5 | **(A)** Summary of the effect of volume differences on mutation cluster assignment using logistic regression. **(B)** Histogram of the effect of three types of mutations, three types are defined by their "contact status" to the DNA. Predicted mutations are subset to the mutations within the structure of 3VD6 in Protein Data Bank.

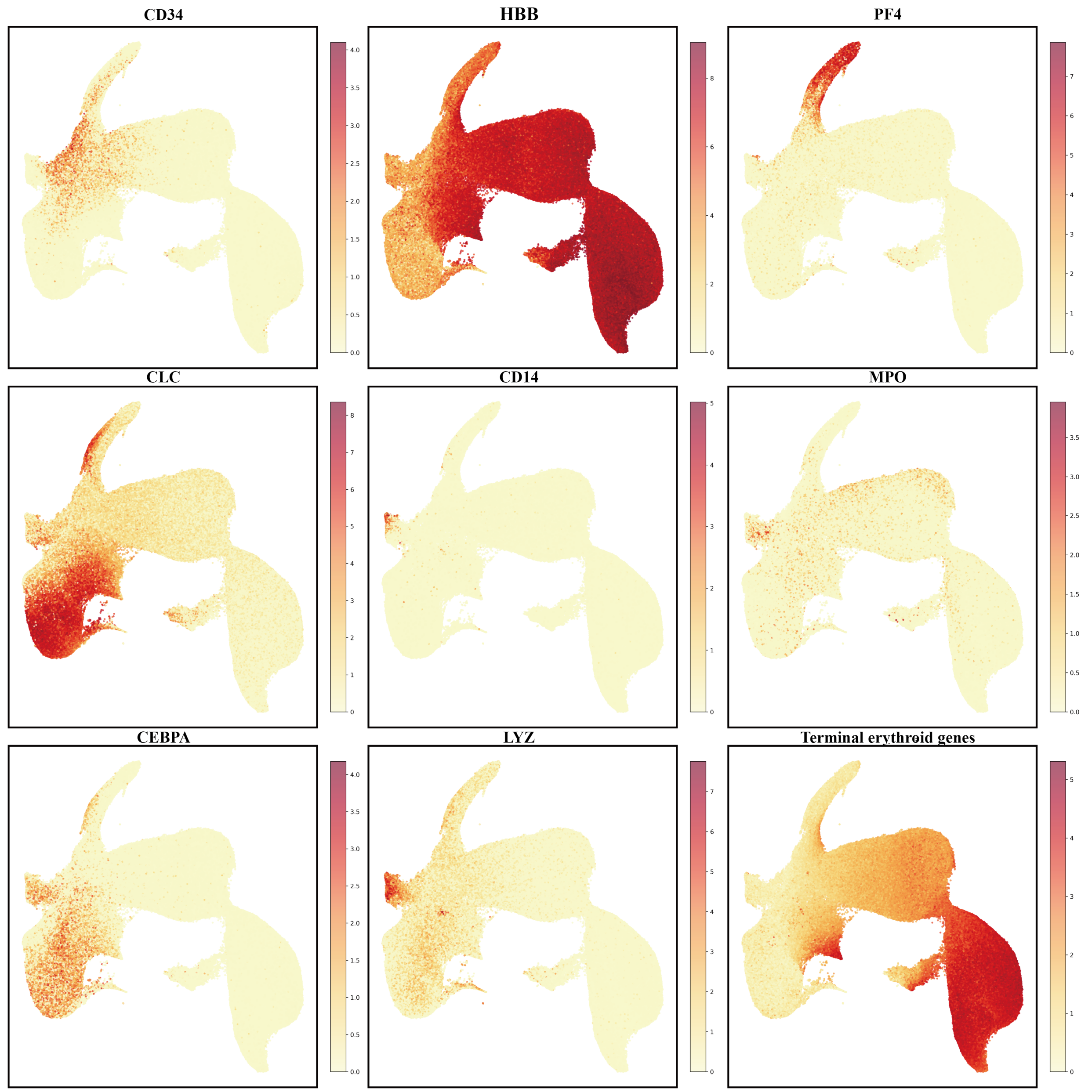

Appendix Figure S6 | UMAP plots of representative markers for each cell lineage within the PerturbNet predicted GATA1 mutations dataset. The terminal erythroid genes represent the mean expression of a set of genes: HBA1, HBA2, HBM, SLC25A37, ALAS2, HEMGN, GYPA, CA1, SOX6, NFIA.

C Supplementary Tables

**Model**	**Median R^2**	**Mean R^2**	**Median R^2 (DEG)**	**Mean R^2 (DEG)**
PerturbNet-normalized	0.943	0.926	0.517	0.421
PerturbNet-raw-counts	**0.946**	**0.930**	**0.662**	**0.573**

Appendix Table S1: Comparison of median and mean R^2 metrics on all genes and the top 50 DEGs between PerturbNet trained on raw counts and PerturbNet trained on normalized expression. The best-performing model for each metric is bolded.

**Component**	**Configuration**
**Input**	Shape: (batch_size, 120, 35)
**Conv1D Layer 1**	`Conv1d(in=120, out=9, kernel_size=9)` + Tanh + BatchNorm1d
**Conv1D Layer 2**	`Conv1d(in=9, out=9, kernel_size=9)` + Tanh + BatchNorm1d
**Conv1D Layer 3**	`Conv1d(in=9, out=10, kernel_size=11)` + Tanh + BatchNorm1d
**Flatten + FC (Encoder)**	`Linear(90 → 196)` + Tanh + Dropout + BatchNorm1d
**Latent Layers**	Two `Linear(196 → 196)` layers for mean and log-variance
**Decoder FC**	`Linear(196 → 196)` + Tanh + Dropout + BatchNorm1d
**Repeat Vector**	Expand to shape `(batch_size, 120, 196)`
**GRU Decoder**	`GRU(input_size=196, hidden_size=488, num_layers=3)` + Tanh
**Final FC + Output**	`Linear(488 → 35)` + Softmax (applied per timestep)
**Output Shape**	`(batch_size, 120, 35)`
**Dropout Rate**	0.0828
**Latent Dim (`z_dim`)**	196
**GRU Hidden Size**	488
**Training Batch Size**	128
**Learning Rate**	1×10^{-4}
**Epochs**	525
**Dataset**	ZINC

Appendix Table S2: ChemicalVAE Model Architecture and Training Parameters

**Component**	**Configuration**
**Input**	Shape: (batch_size, 15,988); one-hot annotation vectors from GO
**Encoder Layer 1**	`Linear(15988 → 512)` + BatchNorm1d + LeakyReLU + Dropout
**Encoder Layer 2**	`Linear(512 → 256)` + BatchNorm1d + LeakyReLU + Dropout
**Latent Mean Layer**	`Linear(256 → 10)`
**Latent Std Layer**	`Linear(256 → 10)`
**Decoder Layer 1**	`Linear(10 → 256)` + BatchNorm1d + LeakyReLU + Dropout(p=0.2)
**Decoder Layer 2**	`Linear(256 → 512)` + BatchNorm1d + LeakyReLU + Dropout(p=0.2)
**Output Layer**	`Linear(512 → 15988)` + Sigmoid
**Dropout Rate**	0.2
**Latent Dim** (`z_dim`)	10
**Training Batch Size**	128
**Learning Rate**	1×10^{-4}
**Epochs**	300
**Dataset**	GO Consortium gene ontology annotations (single and double target genes)

Appendix Table S3: GenotypeVAE Model Architecture and Training Parameters

**Component**	**Configuration**
	**Standard VAE (for normalized data)**
**Input**	Gene expression vectors (normalized data), shape: (batch_size, x_{dim})
**Encoder Layer 1**	`Linear(`x_{dim} `→ 512)` + BatchNorm + LeakyReLU + Dropout(p=0.2)
**Encoder Layer 2**	`Linear(512 → 256)` + BatchNorm + ReLU + Dropout(p=0.2)
**Latent Mean Layer**	`Linear(256 → 10)`
**Latent Scale Layer**	`Linear(256 → 10)` + Softplus
**Decoder Layer 1**	`Linear(10 → 256)` + BatchNorm + LeakyReLU + Dropout
**Decoder Layer 2**	`Linear(256 → 512)` + BatchNorm + LeakyReLU + Dropout
**Output Layer**	`Linear(512 →` x_{dim}`)`
**Latent Dim (z_{dim})**	10
**Dropout Rate**	0.2
**Learning Rate**	1×10^{-4}
**Batch Size**	128
**Epochs**	150
	**scVI Model (for count data, using ZINB likelihood)**
**Model Type**	Variational Autoencoder from `scvi-tools 0.7.1` (Lopez et al. 2018)
**Likelihood**	Zero-inflated Negative Binomial (ZINB)
**Latent Dim (z_{dim})**	10
**Epochs**	700 (default settings)
**Sampling Strategy**	Library size sampled from training set during generation

Appendix Table S4: Cell Representation Network Architecture and Training Parameters

Component	Configuration
**Flow Architecture**	20 invertible blocks, each with:<br>- Alternating affine coupling layer<br>- ActNorm layer<br>- Fixed permutation layer
**Embedding Module**	- Input: conditioning vector<br>- 2 hidden layers, hidden dim = 256<br>- Output dim = 10<br>- Activation: LeakyReLU<br>- Optional BatchNorm (enabled)
**Shared Model Parameters**	`in_channels` = 10, `embedding_dim` = 10, `hidden_dim` = 1024, `hidden_depth` = 2, `activation` = none, `conditioner_use_bn` = True
**Dataset-specific Configuration**	
**Norman et al.**	`conditioning_dim` = 10, `epochs` = 50
**Ursu et al.**	`conditioning_dim` = 1280, `epochs` = 50
**Jorge et al.**	`conditioning_dim` = 1280, `epochs` = 50
**LINCS-Drug**	`conditioning_dim` = 196, `epochs` = 100
**sci-Plex**	`conditioning_dim` = 200, `epochs` = 100
**Training Parameters**	Batch size = 128, Learning rate = 4.5×10^{-6}

Appendix Table S5: Conditional Invertible Neural Network (cINN) Architecture and Training Parameters

**Response: R^2 (highly variable genes)**	**Estimate**	**Std. Error**	**P-value**	**Significance**
Intercept	9.79E-01	1.02E-02	<2E-16	***
number of cells	6.96E-05	1.06E-05	1.39E-10	***
GO term numbers	-3.50E-04	1.10E-04	1.45E-03	**
perturbation type: 0/1	-9.78E-03	6.89E-03	1.56E-01	
perturbation type: 0/2	-1.09E-03	4.29E-03	7.99E-01	
min latent distance	3.01E-02	1.42E-02	3.39E-02	*
number of large effect genes	-2.40E-03	1.82E-04	<2E-16	***
APPROX ADDITIVE	3.47E-03	7.15E-03	6.27E-01	
EPISTASIS	7.04E-03	8.34E-03	3.99E-01	
NEOMORPHIC	3.38E-02	7.56E-03	9.55E-06	***
POTENTIATION	1.08E-02	1.12E-02	3.36E-01	
REDUNDANT	-6.40E-02	9.66E-03	8.62E-11	***
STRONG SYNERGY, DISMILAR PHENOTYPE	2.52E-02	7.20E-03	5.10E-04	***
STRONG SYNERGY, SIMILAR PHENOTYPE	1.44E-02	7.09E-03	4.34E-02	*
SUPPRESSORS	3.44E-02	6.69E-03	3.77E-07	***

Appendix Table S6: Linear regression results for R^2 on highly variable genes (R^2). Significance of the P-values is denoted as follows: *P $<$ 0.05, **P $<$ 0.01, ***P $<$ 0.001.

Response: R^2 (differentially expressed genes)	Estimate	Std. Error	P-value	Significance
Intercept	1.00E+00	6.48E-02	<2E-16	***
number of cells	1.75E-04	6.76E-05	9.97E-03	**
GO term numbers	-8.43E-04	6.96E-04	2.26E-01	
perturbation type: 0/1	-3.50E-02	4.38E-02	4.24E-01	
perturbation type: 0/2	-2.48E-02	2.73E-02	3.63E-01	
min latent distance	1.63E-01	9.01E-02	7.15E-02	
number of large effect genes	-1.71E-02	1.16E-03	<2E-16	***
APPROX ADDITIVE	-4.83E-03	4.54E-02	9.15E-01	
EPISTASIS	-5.59E-02	5.30E-02	2.92E-01	
NEOMORPHIC	1.25E-01	4.80E-02	9.77E-03	**
POTENTIATION	1.88E-01	7.11E-02	8.58E-03	**
REDUNDANT	-2.73E-01	6.14E-02	1.07E-05	***
STRONG SYNERGY, DISSIMILAR PHENOTYPE	2.28E-01	4.58E-02	9.08E-07	***
STRONG SYNERGY, SIMILAR PHENOTYPE	2.34E-01	4.51E-02	3.10E-07	***
SUPPRESSORS	1.31E-01	4.26E-02	2.26E-03	**

Appendix Table S7: Linear regression results for differentially expressed genes (R^2). Significance of the P-values is denoted as follows: *P < 0.05, **P < 0.01, ***P < 0.001.

Response: log[P(depleted)/P(intermediate)]	Estimate	Std. Error	P-value	Significance
Intercept	5.19E+00	1.91E-01	< 2E-16	***
Volume difference	4.40E-03	2.72E-03	1.06E-01	
KD value difference	1.87E-02	3.94E-02	6.35E-01	
Mutation Position: close to DNA-contact residue	-1.13E+00	1.01E+00	2.67E-01	
Mutation Position: Wild Type	-7.89E+00	5.35E+02	9.88E-01	
Mutation Position: DNA-Contact	-2.02E-02	6.19E-01	9.74E-01	

Appendix Table S8: Logistic regression tests the factors associated with cluster assignment between erythroid-depleted and erythroid-intermediate. Significance of the P-values is denoted as follows: *P < 0.05, **P < 0.01, ***P < 0.001.

**Response: log[P(enriched)/P(intermediate)]**	**Estimate**	**Std. Error**	**P-value**	**Significance**
Intercept	-2.37E+00	4.64E-02	$<$ 2E-16	***
Volume difference	-1.93E-03	7.18E-04	7.17E-03	**
KD value difference	5.09E-03	1.03E-02	6.23E-01	
Mutation Position: close to DNA-contact residue	-7.59E-02	1.67E-01	6.50E-01	
Mutation Position: Wild Type	-9.41E+00	1.97E+02	9.62E-01	
Mutation Position: DNA-Contact	-2.16E-01	1.65E-01	1.92E-01	

Appendix Table S9: Logistic regression tests the factors associated with cluster assignment between erythroid-enriched and erythroid-intermediate. Significance of the P-values is denoted as follows: *P $<$ 0.05, **P $<$ 0.01, ***P $<$ 0.001.

**Response: log[P(enriched)/P(depleted)]**	**Estimate**	**Std. Error**	**P-value**	**Significance**
Intercept	2.84E+00	1.98E-01	$<$ 2E-16	***
Volume difference	-6.91E-03	2.93E-03	1.83E-02	*
KD value difference	-1.10E-02	3.98E-02	7.82E-01	
Mutation Position: close to DNA-contact residue	9.93E-01	1.03E+00	3.34E-01	
Mutation Position: DNA-Contact	-2.19E-01	6.44E-01	7.33E-01	

Appendix Table S10: Logistic regression tests the factors associated with cluster assignment between erythroid-enriched and erythroid-depleted. Significance of the P-values is denoted as follows: *P $<$ 0.05, **P $<$ 0.01, ***P $<$ 0.001.

**Amino acid**	**Abbreviations**	**Molecular mass (Da)**	**Number of atoms**	**Volume ($\mathring{A}^3$)**	**Hydropathy index**
Alanine	Ala, A	89	13	88.6	1.8
Arginine	Arg, R	174	26	173.4	-4.5
Asparagine	Asn, N	132	17	114.1	-3.5
Aspartic acid	Asp, D	133	16	111.1	-3.5
Asparagine or Aspartic acid	Asx, B	-	-	-	-
Cysteine	Cys, C	121	14	108.5	2.5
Glutamine	Gln, Q	146	20	143.8	-3.5
Glutamic acid	Glu, E	147	19	138.4	-3.5
Glutamine or Glutamic acid	Glx, Z	-	-	-	-
Glycine	Gly, G	75	10	60.1	-0.4
Histidine	His, H	155	20	153.2	-3.2
Isoleucine	Ile, I	131	22	166.7	4.5
Leucine	Leu, L	131	22	166.7	3.8
Lysine	Lys, K	146	24	168.6	-3.9
Methionine	Met, M	149	20	162.9	1.9
Phenylalanine	Phe, F	165	23	189.9	2.8
Proline	Pro, P	115	17	112.7	-1.6
Serine	Ser, S	105	14	89.0	-0.8
Threonine	Thr, T	119	17	116.1	-0.7
Tryptophan	Trp, W	204	27	227.8	-0.9
Tyrosine	Tyr, Y	181	24	193.6	-1.3
Valine	Val, V	117	19	140.0	4.2

Appendix Table S11: Amino acids with their properties including molecular mass, number of atoms, volume, and hydropathy index. Table is from `https://www.imgt.org/IMGTeducation/Aide-memoire/_UK/aminoacids/abbreviation.html`
