# Supplementary material for: PerturbNet predicts single-cell responses to unseen chemical and genetic perturbations
Source: Mol Syst Biol. 2025 Jul 10;21(8):960–82. doi: 10.1038/s44320-025-00131-3 (PMC12322087; doi:10.1038/s44320-025-00131-3)
Supplement: Supplementary file 12 — Expanded View Figures [file 44320_2025_131_MOESM12_ESM.pdf]

Expanded View Figures

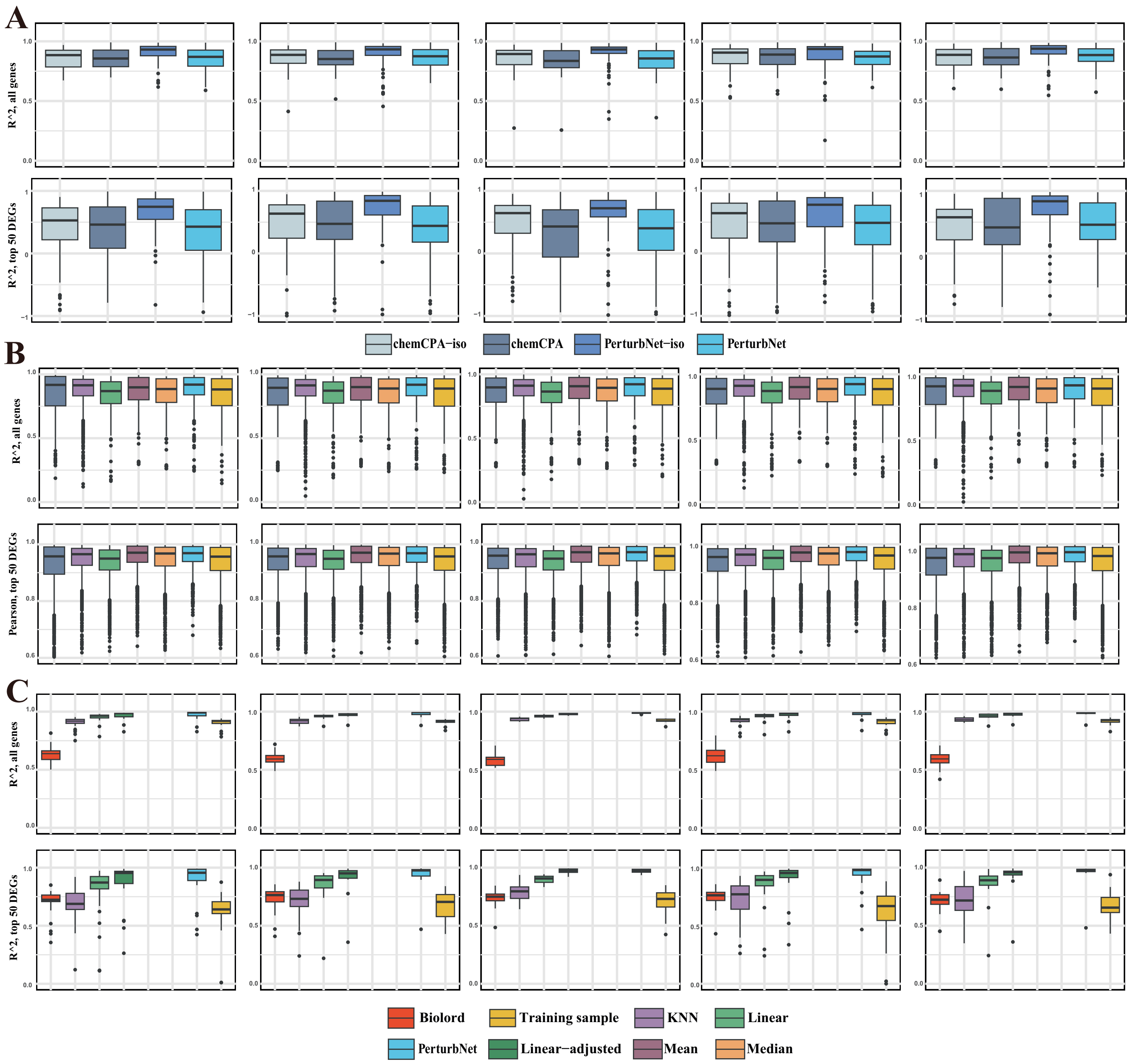

Figure EV1. Box plots of evaluation metrics on the LINCS-Drug and sci-Plex datasets.

(**A**) Comparison between PerturbNet and chemCPA trained with and without stereoisomers. (**B**) Benchmark results on the LINCS-Drug dataset. (**C**) Benchmark results on the sci-Plex dataset. Note: for all box plots in this panel, the box plots show the median (center line), the 25th and 75th percentiles (box bounds), and 1.5× the interquartile range (whiskers). Points beyond the whiskers are plotted as outliers.

 © The Author(s)

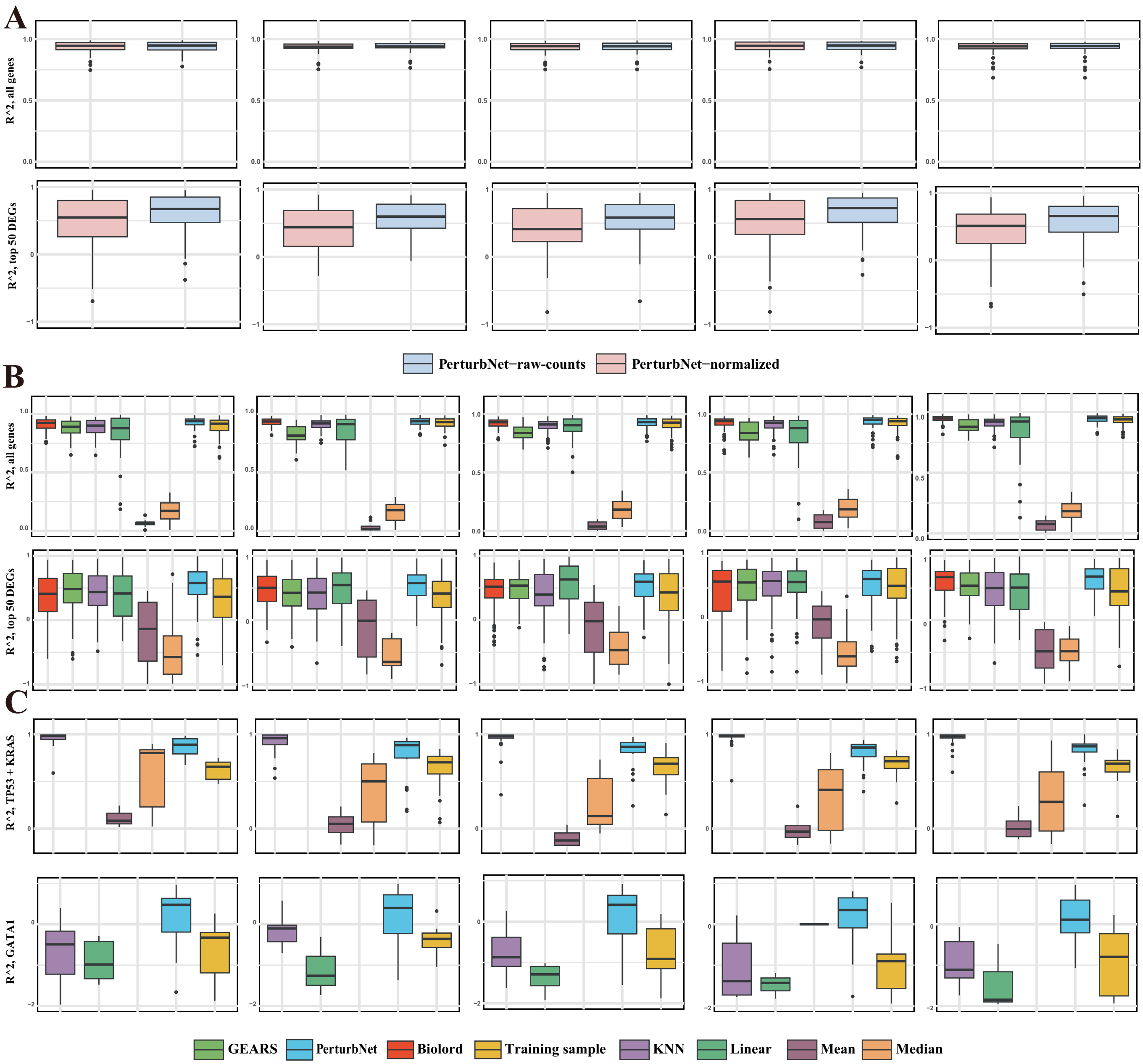

Figure EV2. Box plots of evaluation metrics on the genetic perturbation datasets.

(**A**) Comparison between PerturbNet trained on raw counts and on normalized expression. (**B**) Benchmark results on the Norman et al dataset. (**C**) Benchmark results on the Ursu et al and Jorge et al datasets. Note: for all box plots in this panel, the box plots show the median (center line), the 25th and 75th percentiles (box bounds), and 1.5× the interquartile range (whiskers). Points beyond the whiskers are plotted as outliers.

© The Author(s)

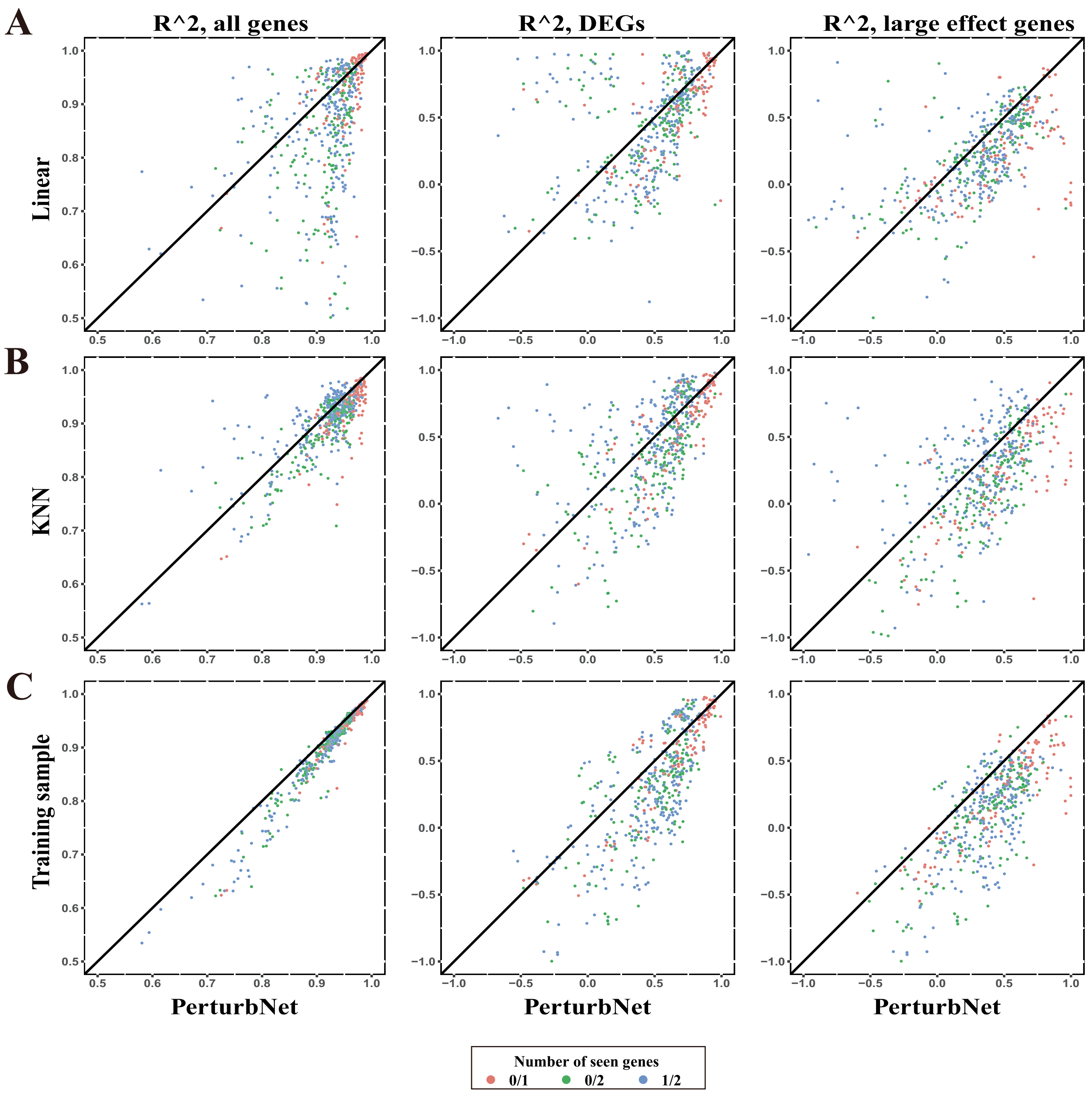

Figure EV3. Scatter plots of R^2 values for unseen genetic perturbations, calculated across all genes, the top 50 differentially expressed genes (DEGs), and large-effect genes from the Norman et al dataset.

Data points are aggregated from all five test splits. Different colors represent the "number of seen genes." Labels such as "0/1" indicate that the test perturbation affects one unseen gene, while "0/2" indicates two unseen genes are perturbed. "1/2" denotes that two genes are perturbed, but one of the target effects has already been observed independently or in combination with other genetic perturbations. (**A**) PerturbNet v.s linear baseline. (**B**) PerturbNet v.s KNN. (**C**) PerturbNet v.s. training sample.

 © The Author(s)

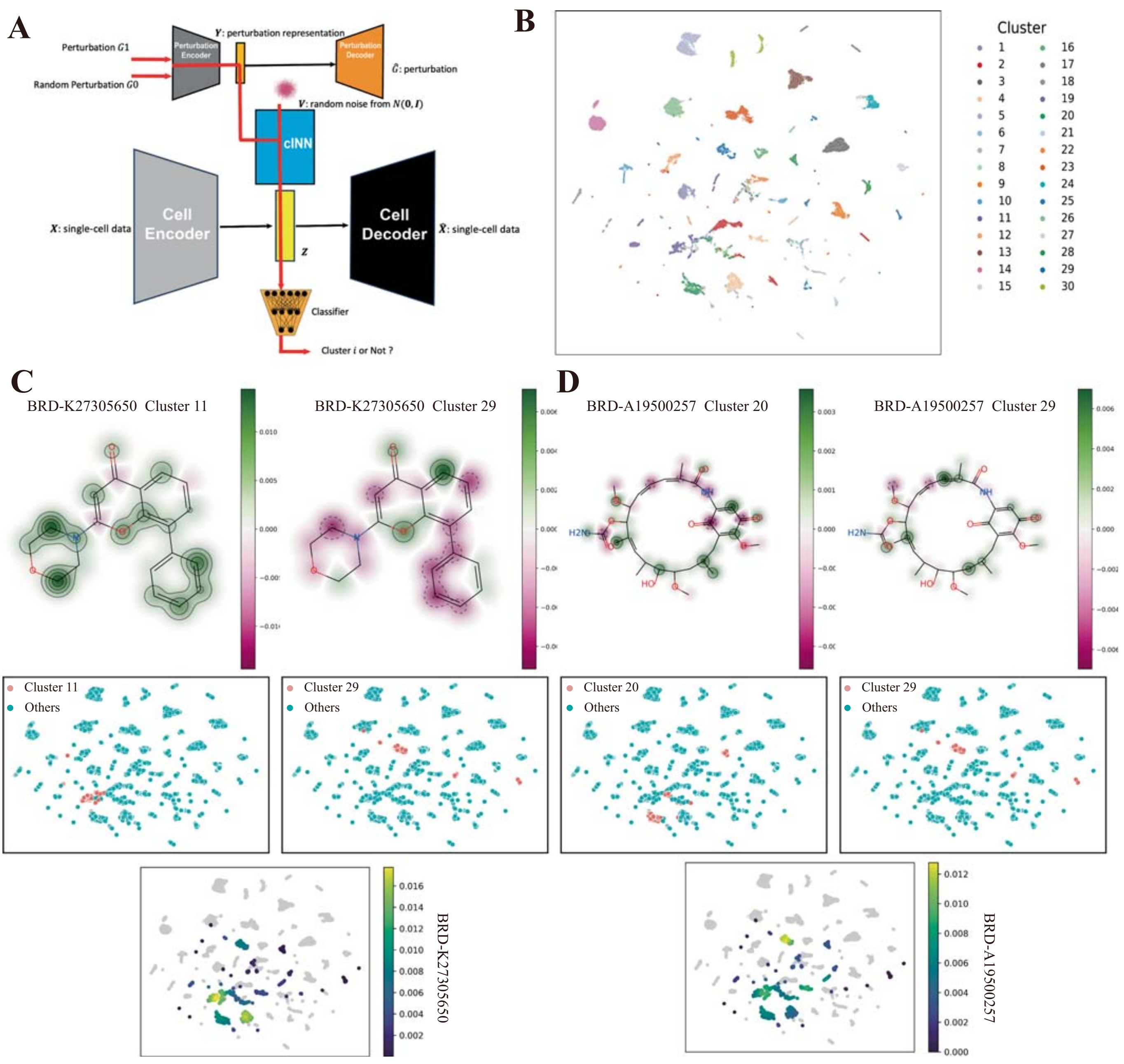

Figure EV4. Attributing cell state shifts to specific features of perturbations.

(**A**) Diagram of approach for attributing perturbation outcomes to specific perturbation features. We attach a binary classifier after the cINN to classify cells into discrete types. By comparing the classification results of an input perturbation and a baseline (random perturbations), we can determine which perturbation features increase classification probability. (**B**) UMAP plots of cells from LINCS-Drug colored by cluster label. (**C**, **D**) UMAP plots of LINCS-Drug with selected clusters and the selected drug colored by attribution scores for each atom. The bottom is the UMAP plot colored by the cell density from selected drugs.

© The Author(s)
